# Induced Hyperproteinemia and Its Effects on the Remodeling of Fat Bodies in Silkworm, *Bombyx mori*

**DOI:** 10.3389/fphys.2018.00302

**Published:** 2018-03-29

**Authors:** Xue-Dong Chen, Yong-Feng Wang, Yu-Long Wang, Qiu-Ying Li, Huan-Yu Ma, Lu Wang, Yang-Hu Sima, Shi-Qing Xu

**Affiliations:** ^1^Department of Applied Biology, School of Biology and Basic Medical Sciences, Medical College, Soochow University, Suzhou, China; ^2^National Engineering Laboratory for Modern Silk (NESER), Institute of Agricultural Biotechnology and Ecology (IABE), Soochow University, Suzhou, China

**Keywords:** animal model, hyperproteinemia, endocrine hormones, fat body, tissue remodeling

## Abstract

Hyperproteinemia, which is characterized by an abnormally elevated plasma protein concentration (PPC), is a high-mortality, metabolic complication associated with severe liver and kidney disease. It is difficult to clinically distinguish the difference between the impacts of primary diseases and hyperproteinemia on tissues and organs, and there are no available animal models of hyperproteinemia. Here, we constructed an animal model of hyperproteinemia with a controllable PPC and no primary disease effects in the silkworm *Bombyx mori* that has attracted interest owing to its potential use in the pathological analysis of model animals. Silkworm have an open circulatory system in which each organ is directly immersed in hemolymph. The fat body (FB) of a silkworm, as a major organ for nutrient storage and energy metabolism, can effectively reflect hyperproteinemia-induced metabolic abnormalities in damaged visceral tissues. A pathogenesis study showed that hyperproteinemia attenuated cell autophagy and apoptosis by attenuating an endocrine hormone, thereby preventing FB remodeling during metamorphosis. Meanwhile, hyperproteinemia increased oxidative stress in the FB and resulted in a dysfunction of amino acid conversion. Supplementation with exogenous 20-hydroxyecdysone effectively mitigated the hyperproteinemia-mediated inhibition of FB remodeling.

## Introduction

Many diseases show hyperproteinemia with an abnormally elevated plasma protein concentration (PPC) (Nordon, 1974; Bernardini et al., 2009; Safavi and Honarmand, 2009); these include cirrhosis (Bergstedt and Lingen, 1957; Schneiderbaur, 1958), multiple myeloma (Foord, 1935; Gutman et al., 1941), angiosarcomatosis (Sonnet et al., 1961), metabolic acidosis (Wang et al., 1986), nephropathy (Salvesen, 2009), mammalian peritonitis (Riemer et al., 2016), chronic lymph flow (Manning, 1998a), abdominal abscess (Magid, 2006), and nematode infections (Adeyeba, 1985). Because it is difficult to distinguish between the impacts of hyperproteinemia and primary diseases or infections on an organism, previous reports have only focused on a comparison of basic data that show the impact of metabolic diseases, such as hypoalbuminemia, hyperglycemia, or hyperlipidemia, on the physical and chemical properties of blood (Bartelheimer and Schwartzkopff, 1954; Manning, 1992, 1998b; Bernardini et al., 2009). There are almost no reports of hyperproteinemia disease models and any relevant pathological mechanisms. Therefore, clinical research of hyperproteinemia, as well as drug development, has been stagnant for a long time, and there are no reliable hyperproteinemia disease models or modeling methods.

This study aimed to establish a reliable hyperproteinemia disease model and use it to assess the effects of hyperproteinemia on important metabolic tissues. To this end, we chose an invertebrate model animal, the silkworm *Bombyx mori* (*B. mori*) (Xia et al., 2009; Tabunoki et al., 2016; Xing et al., 2016). During silkworm larval–pupal metamorphosis, there are a series of developments and changes of multiple tissues and systems that are similar to those in mammalian embryos or adolescents (Dong et al., 2007; Liu et al., 2015, 2016), among which the most typical events are tissue remodeling, such as the metabolic reorganization of the fat body (FB) (Liu et al., 2009; Kaneko et al., 2011; Xia et al., 2014).

The silk gland (SG) of *B. mori* grows rapidly, and it exhibits some similarities to some rapidly growing tumors (Wang et al., 2016; Xing et al., 2016). During the entire larval stage, the size of the SG increases 10,000-fold, and its cellular metabolism is extremely fast and cells do not undergo apoptosis, while nuclear DNA is replicated several times in SG cells and the DNA content increases 200,000–400,000-fold within 3.5 weeks. The synthesized silk proteins in the SG account for more than 30% of the body weight of the organism (Ji et al., 2013; Xia et al., 2014). Thus, we hypothesized that a rapid release of a large amount of protein from the SG into the hemolymph would lead to a sharp increase in the PPC.

The FB of *B. mori* is an organ analogy to vertebrate adipose tissue and liver, and it functions as a major organ for nutrient storage and energy metabolism. The FB undergoes a developmental remodeling process during larval–pupal metamorphosis, which is characterized by a massive destruction of obsolete larval tissues by programmed cell death and the simultaneous growth and differentiation of adult tissues from small clusters of progenitor cells (Liu et al., 2009; Kaneko et al., 2011). FB development and function are largely regulated by insulin, as well as ecdysteroid and nutritional signals, including the proto-oncogenes and tumor suppressors, in these pathways (Warbrick-Smith et al., 2006; Liu et al., 2009). Hence, choosing the FB as a target tissue for research can provide a wealth of information on the effects of hyperproteinemia on visceral tissues during metamorphosis, and it may help further the exploration of its interaction with endocrine hormone signaling pathways.

## Materials and methods

### Preparation of animals

The *Dazao* strain of *B. mori*, a classical genetic strain, was used in this study. The larvae were reared on fresh mulberry (*Morus alba* var. multicaulis) leaves at 25°C with a 12 h light/12 h dark photoperiod until they grew into mature larvae, namely the wandering stage, at which point they were ready to enter the subsequent spinning stage and complete their metamorphosis. Then, the animals were kept at the same temperature and light conditions as larvae, but were not fed any bait until they grew into adult moths.

The animal model (AM) of hyperproteinemia was induced in wandering stage larvae by covering the spinnerets with low-melting-point paraffin wax. To observe the influence of an exogenous hormone on the hyperproteinemia-mediated delay in FB remodeling, the larvae were injected with 4 μg of 0.4 μg/μL 20-hydroxyecdysone (20E; Sigma-Aldrich, St. Louis, MO, USA) 24 h after the induction of hyperproteinemia (AM+20E) and the larvae of negative AM control were injected with the same amount of dimethyl sulfoxide. Then, the larvae were kept under natural conditions, like the control (CK) group, until they grew into adult moths.

To observe the morphology of the silkworm, the cocoon shells in the CK group were peeled off every 12 h until 48 h after spinning. This treatment also ensured the consistency of the micro-environment among the silkworms that spun cocoons (the CK group) and those that did not (the AM and AM+20E groups).

### Individual metamorphosis survey

Beginning at the mature larval stage, the development of the AM, AM+20E, and CK groups was investigated every 12 or 24 h, during the wandering, spinning, pre-pupal, semi-pupal, pupal, and adult stages. Meanwhile, animal deaths were counted. A large number of larvae (*n* ≥ 90) constituted the AM and CK groups used to assess the effects of the elevated PPC on animal development and viability. To further investigate the mitigating effects of 20E on the hyperproteinemia-induced impairment of FB remodeling, ≥ 32 larvae constituted CK, AM and AM+20E groups.

### Determination of the PPC

Equal volumes of hemolymph were collected from three individual silkworms after cutting the anal horns of the larvae and the abdominal epidermis of the pupae on ice and combined to form samples. Then, the hemolymph was centrifuged at 1,400 × *g* for 15 min at 4°C. Twenty microliters of supernatant was used to measure the PPC with a BCA protein assay kit (Beyotime, Jiangsu, China). We measured each sample three times (*n* = 3). After hemolymph sampling, the SG samples were isolated from the above silkworms on ice and used for morphological observations.

### Observation of FB dissociation

To observe the connections of FB cells, the cytomembrane was stained with 1,1′-dioctadecyl-3,3,3′,3′-tetramethylindocarbocyanine perchlorate (DiI) (Beyotime, Jiangsu, China), which was reconstituted with dimethyl sulfoxide. The FB samples were isolated every 24 h in CK and AM groups, washed using normal saline (0.7% NaCl), and then quickly placed into a 10 μM DiI staining solution for 15 min at room temperature. After washing three times with normal saline, the FB was observed under a fluorescence microscope (Olympus SZX16, Tokyo, Japan).

### Hematoxylin–eosin (HE) staining and immunohistochemistry

The tissue section staining followed the method of Fang et al. (2014) and Ji et al. (2013). The FB samples were isolated every 24 h in CK and AM groups, and fixed immediately in 4% paraformaldehyde. After serial dehydration in ethanol, and inducing transparency using xylene, the samples were embedded in paraffin blocks, and then cut into 5–10-μm thick sections. The sections were dewaxed using xylene and rehydrated in an ethanol series, and then stained with HE. The immunohistochemistry followed the method of Wang et al. (2016) and Ji et al. (2013). The dewaxed and rehydrated sections were subjected to an antigen unmasking procedure by heating in sodium citrate buffer for 15 min, followed by cooling. Then, the sections were incubated with an anti-human cleaved-caspase-3 antibody (Cell Signaling Technology, Beverly, MA, USA) overnight at 4°C. After blocking for 40 min with a blocking solution, followed by washing three times with phosphate-buffered saline with Tween 20 (PBST), the sections were incubated for 30 min with a fluorescein isothiocyanate-conjugated secondary antibody (anti-rabbit) at 37°C. The sections were washed three times with PBST and then stained for 10 min with 4′,6-diamidino-2-phenylindole (Beyotime, Jiangsu, China). Reference sections were treated with rabbit negative serum. All sections were visualized under an Olympus BX51 fluorescence microscope.

### Lysosomes and reactive oxygen species (ROS) staining

Beginning at the mature larval stage, FBs were isolated from the silkworms of the CK and AM groups every 24 h and used for subsequent lysosomes and ROS staining. Briefly, the lysosomes in the cells of freshly isolated FBs were stained with 50 nM LysoTracker Red (Beyotime, Jiangsu, China). The samples were washed three times with normal saline (0.7% NaCl) and then incubated for 30 min at 25°C in 2 mL of staining liquid (LysoTracker Red DND-99). After staining, the samples were washed three more times. The ROS levels were measured with the Reactive Oxygen Species Assay Kit (Beyotime, Jiangsu, China). The FB were collected in normal saline (0.7% NaCl) to avoid exposure to air, then quickly placed into the ROS-staining solution for 30 min, and washed for 5 min with saline in the dark. The red fluorescence from the lysosomes and green fluorescence from the ROS were observed with a fluorescence microscope (Olympus BX51).

### Gene expression analysis

Total RNA was isolated from each FB sample, which were mixtures of FBs collected from three individual silkworms, using the RNAiso Plus Kit (TaKaRa, Dalian, China) at 24, 48, 72, and 96 h after reaching the mature larval stage. cDNA was synthesized with the PrimeScript™ RT (Perfect Real Time) Reagent Kit with gDNA Eraser (TaKaRa), according to the manufacturer's instructions. Quantitative real-time reverse transcription–PCR (qRT-PCR) was used to analyze the mRNA transcript levels of the ecdysone receptor (*BmEcR*), early transcription factor 74a (*BmE74A*), cathepsin B (*BmCatB*), cathepsin D (*BmCatD*), autophagy 6 (*BmAtg6*), autophagy 8 (*BmAtg8*), and apoptotic death regulator Nedd2-like caspase (*BmDronc*) genes, while the *B. mori* α-tubulin gene was selected as the internal control. PCR was performed in a 20 μL reaction volume using the ABI StepOnePlus™ Real-Time PCR System (Ambion, Foster City, CA, USA) and the fluorescent dye SYBR Premix Ex Taq (TaKaRa, Dalian, China), according to the manufacturers' instructions and the method of Ji et al. (2013). The reaction conditions for the qRT-PCR were as follows: 95°C for 1 min and 40 cycles of 95°C for 15 s, 60°C for 1 min. The primers used in this study are listed in Table S1.

### Determination of free amino acids

The contents of free amino acids in FBs at 24, 96, and 192 h after the mature larval stage were determined with high-performance liquid chromatography (HPLC) and quantified by an external standard method. Each FB sample (0.50 g) was a mixture obtained from 10 individuals and was used for the amino acid determination. Three samples were measured in each group. Liquid nitrogen was added, and the mixture was quickly ground, and then 1 mL of 5% trichloroacetic acid was added. The mixture was collected into a centrifuge tube and centrifuged at 13,200 × *g* for 15 min. The supernatant was filtered through a 0.22-μm membrane filter and assayed with HPLC (Ag1100, Agilent, Palo Alto, CA, USA). Pre-column derivatization with o-Phthalaldehyde and 9-fluorenylmethyl chloroformate was used to determine the free amino acid contents. The HPLC used an ODS HYPERSIL column (250 × 4.6 mm, 5 μm). The mobile phase A was 48 mM NaAc solution, consisting of 225 μL triethylamine and 5 mL tetrahydrofuran per L. The mobile phase B was 0.24 M NaAc solution: acetonitrile: methanol = 1: 2: 2. The detection wavelengths were 338 and 262 nm.

### Biochemical assays

Each FB sample (0.1 g), which was a mixture from 10 larvae of the CK or AM group, was obtained in triplicate for the biochemical assays. Three samples were measured in each group. The activities of glutamic–pyruvic transaminase (GPT), glutamic oxaloacetic transaminase (GOT), catalase (CAT), glutathione S-transferase (GST), and superoxide dismutase (SOD) were measured using their respective assay kits (Comin, Suzhou, China) according to the manufacturer's instructions. The absorbances of all samples were measured using a microplate spectrophotometer (Eon, BioTek Instruments Inc., Winooski, VT, USA).

### Data analyses

All outcome data were analyzed using Statistical Package for Social Sciences (SPSS; Version10.0). A one-factor ANOVA, followed by a post hoc Newman–Keuls comparison, was performed on the gene expression level, enzymatic activity, free amino acid content, and the PPC. Data are presented as mean ± SEM and significance was accepted at *p* < 0.05 (^*^) and *p* < 0.01 (^**^).

## Results

### Animal model (AM) of hyperproteinemia

**Figure 1:**
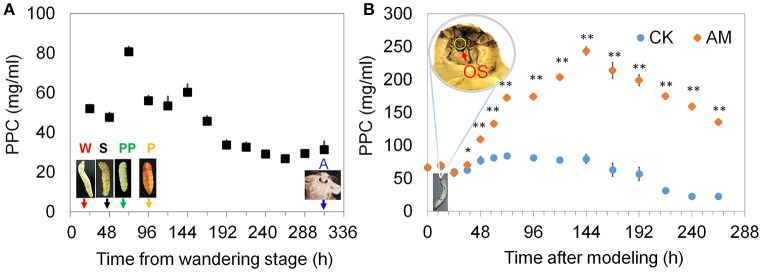
Changes in the plasma protein concentration (PPC). **(A)** The 24 h interval PPCs from the wandering to the adult stage in the control (CK) group. **(B)** The 12 or 24 h interval PPCs in the animal model (AM) of hyperproteinemia and the CK groups. The spinneret of mature *B. mori* larvae in the AM group was covered with low-melting-point paraffin wax at the wandering stage. Then, the larvae were maintained at 25°C with a 12 h light/12 h dark photoperiod without feeding or treatment, like the larvae in the CK group, and the PPC was monitored throughout development. Each hemolymph sample for surveying PPC consisted of an equal volume of hemolymph from three individual silkworms and was measured three times. W, the wandering stage; S, the spinning stage; PP, the pre-pupal stage; P, the pupal stage; A, the adult stage; OS, orifice of the spinneret. ^*^ and ^**^ indicate significant differences between the two groups at *P* < 0.05 and *P* < 0.01, respectively (*n* = 3).

It has been reported that structural proteins are released from a degenerating SG into the open circulatory system, along with non-released fibroin from the glandular cavity, which accounts for 2.5% of the silkworm's body weight (Jia et al., 2007; Ji et al., 2013; Xia et al., 2014; Wang et al., 2015). Therefore, we detected changes in the PPC in silkworms from the wandering to the adult stages. The data showed that this physiological process leads to a transient increase in the PPC, but that the PPC was restored to its pre-elevated level after 24 h (Figure 1A). If the fibroin in the SG is prevented from being released outside the silkworms by covering the spinneret of *B. mori* larvae with low-melting-point paraffin wax at the wandering stage, the fibroin in the glandular cavity that accounts for 30% of the body weight is also released into the circulatory system in the subsequent metamorphosis during the physiological degradation of the SG. Figure 1B shows that the PPC level increased gradually from 48 to 144 h after blocking the spinneret, which is consistent with the SG degradation process (Figure S1), indicating that SG-released proteins lead to an increased PPC during SG degradation. Although the PPC level in the AM group was down-regulated from 48 to 144 h, similar to the CK group, the PPC level in the AM group was much higher than that in the CK group. Therefore, the follow-up experiments in this study focus on the effect of the high PPC on tissue remodeling during 48–192 h of development.

**Figure 2:**
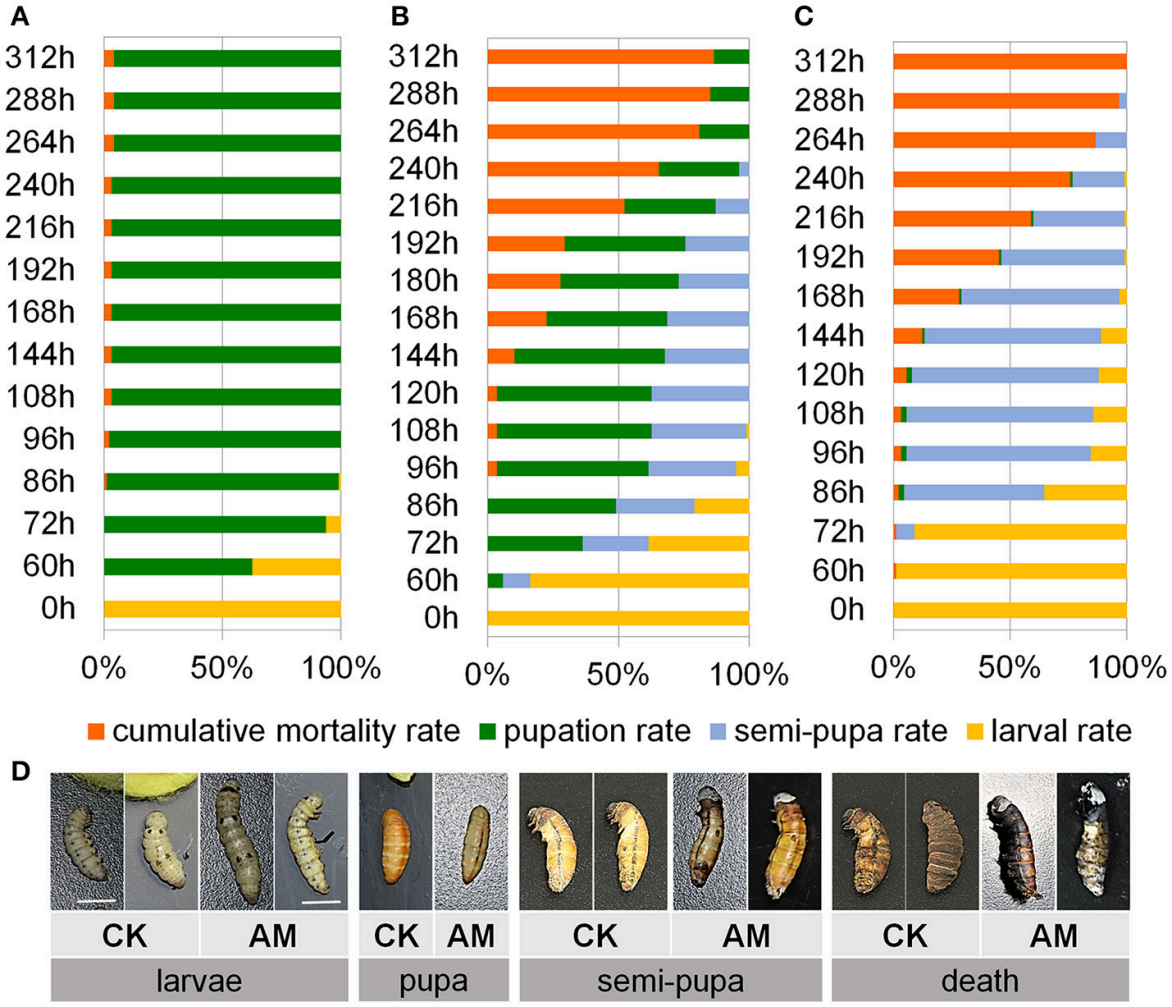
Impact of hyperproteinemia on development and survival. Index of development and survival expressed in terms of the cumulative mortality, pupation rate, semi-pupa rate, and larval rate in the **(A)** negative control (CK) group (*n* = 92 individuals) and **(B,C)** the animal model (AM) of hyperproteinemia group (*n* = 90 individuals). **(B)** At 24 h after the wandering stage, the spinneret was covered with paraffin wax after spinning silk for 17 h, and approximately 50% of the silk protein remained in the silk gland (SG), while **(C)** at the wandering stage, the spinneret was completely impermeable and all the silk proteins remained in the SG. The larval spinnerets of *B. mori* in the CK group were not covered, and approximately 8% of the silk protein remained in the SG. **(D)** Morphological comparisons of silkworm development between the AM of hyperproteinemia and the CK group. Bars are 1 cm in **(D)**. To observe the morphology of the silkworm, the cocoon shells in the CK group were peeled off every 12 until 48 h after spinning. This treatment also ensured the consistency of the micro-environment among the silkworms that spun their cocoons in the CK group and those that did not in the AM group.

To assess the effects of the elevated PPC on animal development and viability, we investigated animal development and survival after hyperproteinemia was induced. Figure 2A shows that although a physiological transient elevation of the PPC occurred during larval–pupal metamorphosis of the CK group, 62.5% of the silkworms went through metamorphosis to the pupal stage 60 h after the wandering stage, and all surviving individuals completed metamorphosis after 96 h. The metamorphosis mortality rate was only 2.1%.

The SG was treated using the method shown in Figure 1 to induce a high PPC in silkworms. Figure 2C shows that no individuals developed into pupae at 60 h after covering the spinneret completely with paraffin wax, and only 2.2% developed into pupae at 96 h, while > 50% of the surviving silkworms remained in the semi-pupa state at 192 h. These results show that a high level PPC blocks silkworm metamorphosis, as the majority of surviving individuals could not complete metamorphosis. Additionally, although the metamorphic mortality rate of the silkworms with blocked spinnerets was only approximately 3%, which was close to that of the CK group, all the other individuals died during the pupal phase. Figure 2D shows the morphology of the silkworm.

To verify the reliability of the AM, approximately 50% of the silk protein was confined in the SG by covering the spinneret with paraffin wax after spinning silk for 17 h to establish a mild case of hyperproteinemia. Figure 2B shows that 7.5% of the individuals developed into pupae 60 h after the wandering stage, and 60% developed into pupae at 96 h, while at 192 h, although more than 25% of the surviving individuals remained in the half-pupation phase, 44.2% of the individuals completed pupation. The blockage of metamorphosis was significantly mitigated compared with the treatment shown in Figure 2C, and that the development of silkworm larvae/pupae and their death rate are closely related to the PPC and the length of time at which the high PPC was maintained.

### Hyperproteinemia blocks FB remodeling

**Figure 3:**
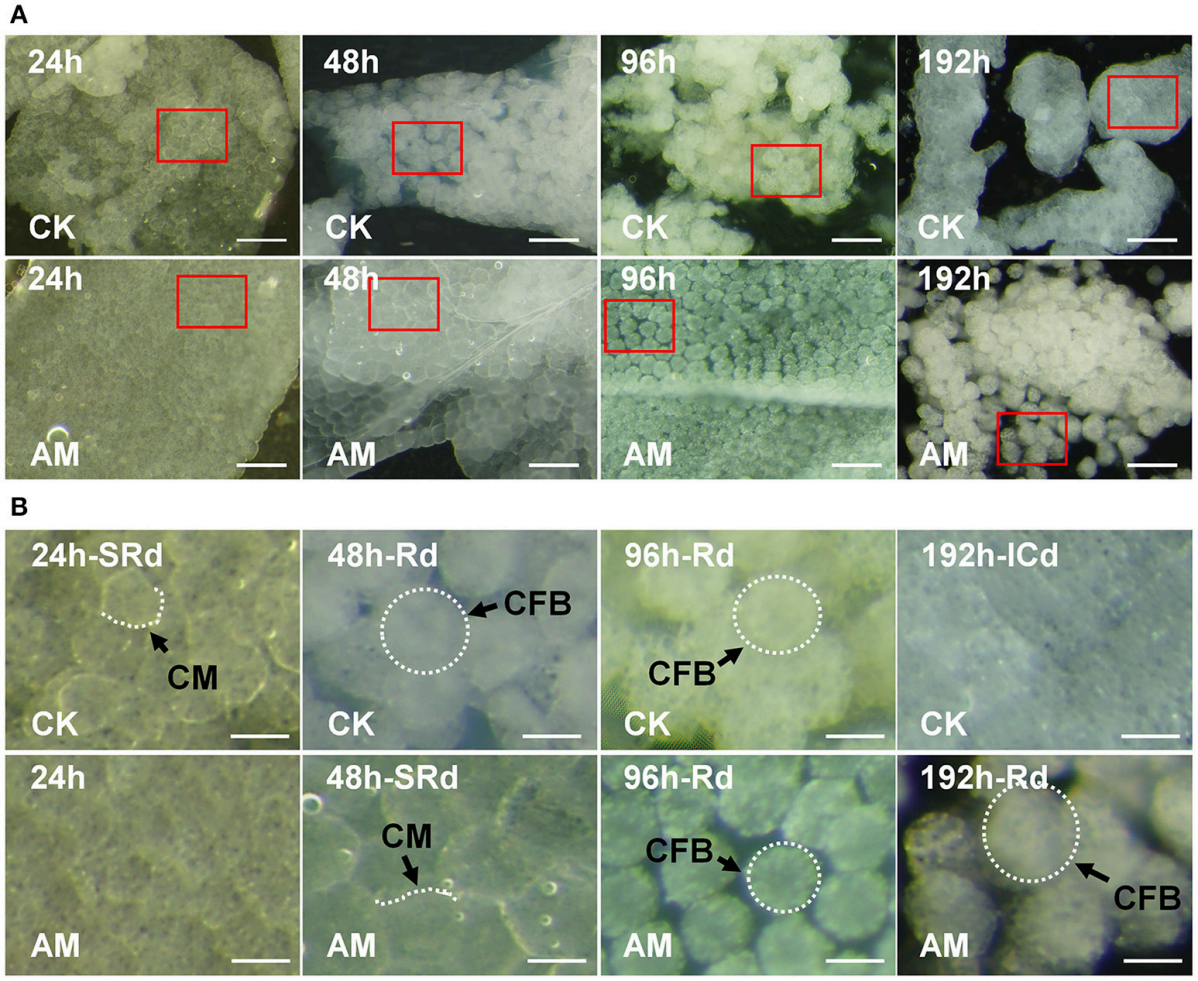
Influence of hyperproteinemia on fat body (FB) remodeling. **(A)** Histomorphology of the FB during larval–pupal metamorphosis. **(B)** The amplified images in **(A)** are marked with red frames. *Bombyx mori* larvae were treated and sampled as described in the Figure 1. The spinneret was covered with paraffin wax for 24, 48, 96, and 192 h. CK, the control group without treatment; AM, the animal model of hyperproteinemia group; CM, cell membrane; FBC, FB cell; SRd, start of FB remodeling; Rd, FB remodeling; ICd, initially complete remodeling of the FB. The bar is 20 μm in **(A)** and 5 μm in **(B)**.

During the normal silkworm FB remodeling process (Figure 3A), at 24 h after the wandering stage, the closely connected cells start to become poorly connected. At 48 h, the larval FB starts to dissociate, the basement membrane ruptures, the cell connections begin to loosen, and the cells become separated from the tissue. At 96 h, the FB is completely dissociated, and the cells are in the process of autophagy, and at 196 h, the adult FB forms and FB cells are connected closely to each other, whereas in the AM of hyperproteinemia, FB remodeling is blocked, or at least significantly slowed down. The enlargement in Figure 3B shows that FB cells of AM silkworms maintained a close association with the larval FB at 24 h of the wandering stage, while at 96 h, the basal membrane became thinner and the FB cells became loosely connected, and at 192 h, the FB completely dissociated. Figure S2 shows the continuous FB morphological changes within 240 h after the wandering stage, which further proves that there is no adult FB 240 h after hyperproteinemia was induced.

**Figure 4:**
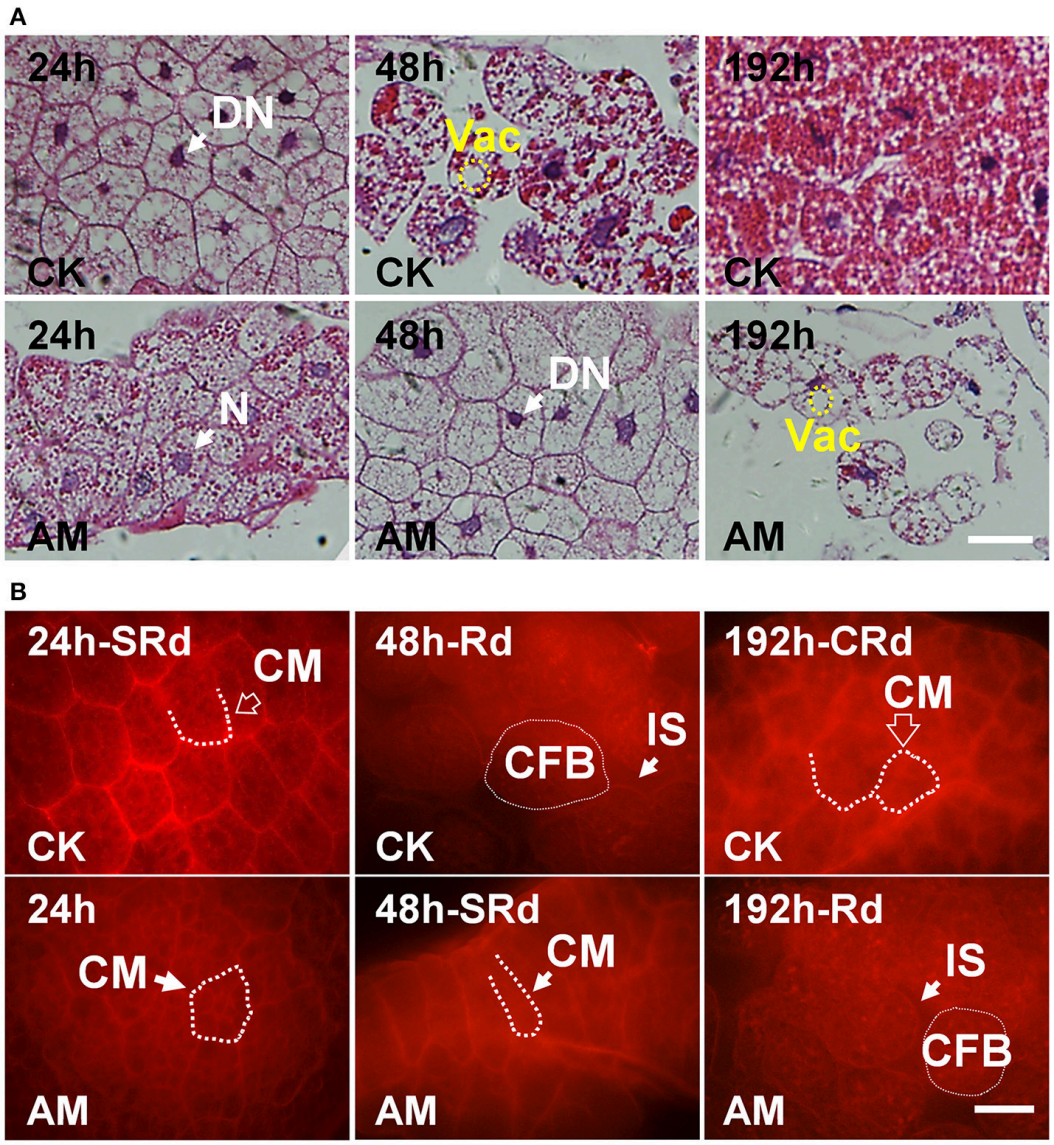
**(A)** Hematoxylin-eosin staining and **(B)** cell membrane staining of the silkworm fat body (FB). *Bombyx mori* larvae were treated and sampled as described in the Figure 1. CK, the control group; AM, animal model of hyperproteinemia. FBs were analyzed at 24, 48, and 192 h after hyperproteinemia was induced. N, normal nuclei; DN, dense nuclei, CM, cell membrane; Vac, vacuolation; CFB, FB cells; IS, intercellular space; SRd, start of FB remodeling; Rd, remodeling of FB; CRd, complete remodeling of FB. The bar = 5 μm.

To confirm the time course of larval FB remodeling, we used hematoxylin and eosin (HE) staining to observe the details of the FB paraffin sections. The results at 24, 48, 192, (Figure 4A), and 240 h (Figure S3) showed that the normal silkworm FB remodeling process was complete within 196 h after the wandering stage, while the FB degradation in the larvae of AM silkworms was significantly slower. No adult FB formed at 216 h after the wandering stage. This is highly consistent with the observations of fresh tissues, as shown in Figure 3.

Furthermore, we stained FBs at three different time points after hyperproteinemia was induced to investigate the intercellular connections, as shown in Figure 4A. At 24 h after hyperproteinemia was induced, the cell membrane of the FBs in both the CK and AM silkworms were well-organized and were clearly stained red, which is indicative of close connections. At 48 h after hyperproteinemia was induced, the intercellular connections of FB cells in the CK group decreased and the morphology of the cells changed from polygonal to oval, indicating that the interconnection of the cells decreased or disappeared, suggesting that the cells began to dissociate. Meanwhile, the cell membrane of FB cells in the AM group was well-stained and exhibited tight intercellular connections, and irregular polygons appeared in the cells, indicating that the cells had not dissociated. At 192 h, the FB tissues in the CK group completely re-formed a relatively stable tissue morphology, and the cells showed tightly connected polygons. Meanwhile, the intercellular membrane of the AM cells was not tightly connected and the cells were dissociating.

### Effects of hyperproteinemia on the metabolism of free amino acids in tissues

**Figure 5:**
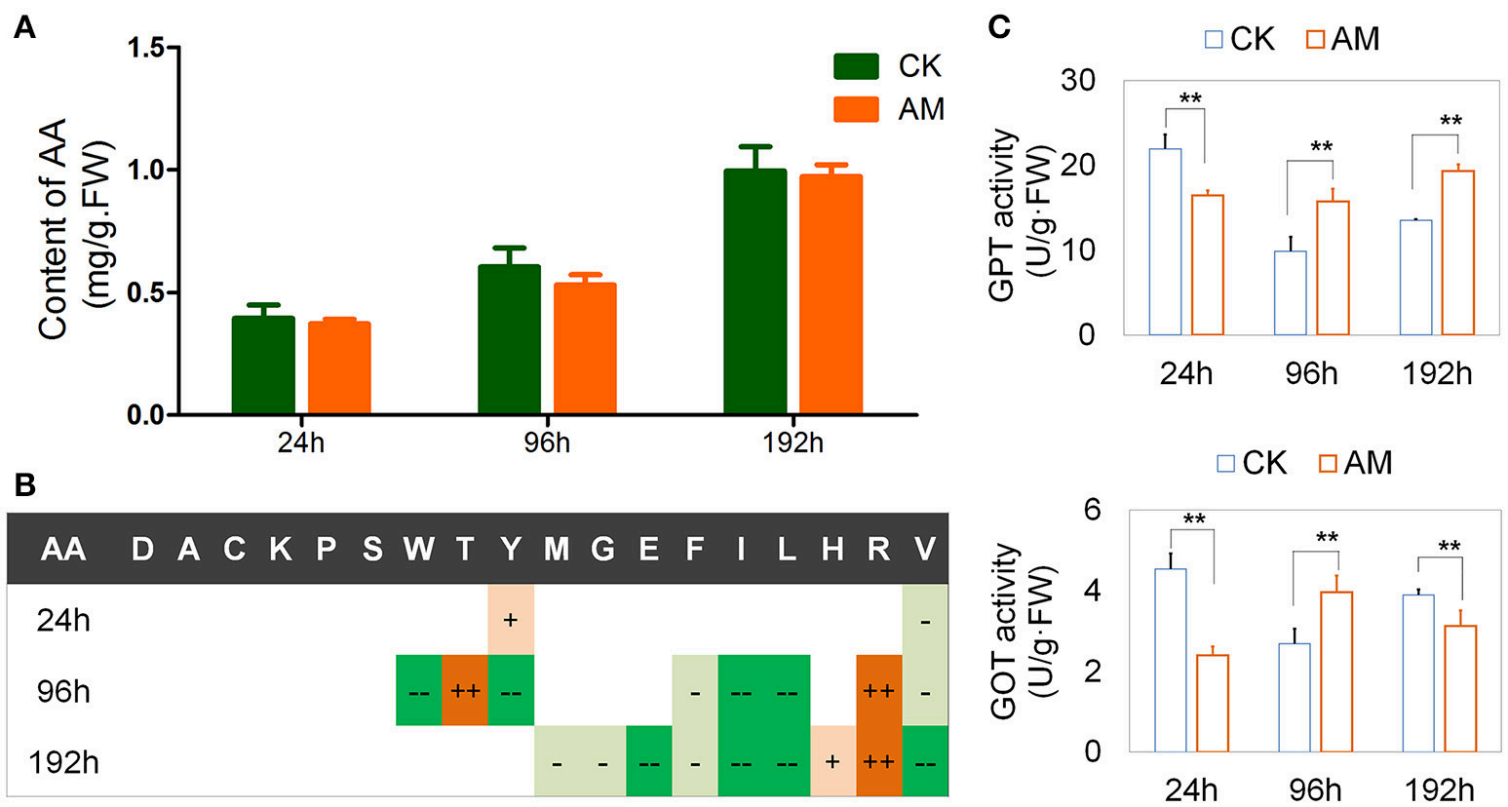
Effects of hyperproteinemia on free amino acid contents and enzymatic activities. *Bombyx mori* larvae were treated as described in the Figure 1. Each FB sample was a mixture obtained from 10 individuals and was used for the amino acid determination (sample weight 0.50 g) and the biochemical assays (sample weight 0.10 g). At each time-point, three samples were measured in each group. **(A)** Total contents of free amino acids in the fat body and **(B)** significant statistical differences between the animal model (AM) of hyperproteinemia and the control group without treatment (CK). **(C)** Activity of glutamic-pyruvic transaminase (GPT) and glutamic oxaloacetic transaminase (GOT) 24, 96, and 192 h after hyperproteinemia was induced. In **(B)**, + and − symbols indicate that the contents of free amino acids in the AM group were higher and lower, respectively, than those in the control group. Single + or − symbols indicate *P* < 0.05, and two ++ or −− symbols indicate *P* < 0.01 (*n* = 3). In **(C)**, ^**^ indicates *P* < 0.01 (*n* = 3). The absolute values about **(B)** was shown in Table S2.

Figure 5A shows that the free amino acid contents in the FBs of the CK and AM groups were highly similar and did not exhibit any significant differences. Although free amino acids level increased 192 h after hyperproteinemia was induced, it could be interpreted as the dissociation of proteins due to autophagy, or other dead cells during tissue reformation. A further analysis of the free amino acid composition showed that the Tyr level in the AM group was transiently elevated at 24 h, decreased at 96 h, and then returned to the level of the CK group at 192 h. The Trp level in the AM group decreased at 96 h. In contrast to Trp, the Thr level in the AM group was transiently elevated at 96 h. However, they both returned to the level of the CK group at 192 h. There were no significant differences in the Asp, Ser, Ala, Lys, and Pro levels between the AM and CK groups, indicating that the FB has a strong capability to regulate several amino acids (Figure 5B and Table S2).

An amino acid composition analysis also showed that the Arg level in the AM group was significantly higher than in the CK group after 96 h, indicating that Arg metabolism was hindered (Figure 5B and Table S2). In some animals, including silkworms and mammals, Arg metabolism can transform blood ammonia into urea and maintain the balance of nitrogen (Isoe and Scaraffia, 2013), in addition to being involved in immune regulation and defense (Dzik, 2014). In this case, the abnormal increase of the Arg level in the FB of hyperproteinemic silkworms suggests that the nitrogen balance and other functions have been affected.

Additionally, the free amino acid of Ile, Leu, and Val, which are branched-chain amino acids (BCAAs), in the FB of silkworms in the AM group were significantly lower than those in the CK group. It has been reported that BCAAs are required for the transformation of protein, sugars, and lipids through ketogenesis, gluconeogenesis, and the tricarboxylic acid cycle. A lack of BCAAs can lead to metabolic diseases (Higuchi et al., 2011; Burrill et al., 2015; Green et al., 2016; Lerin et al., 2016). Therefore, it is suggested that hyperproteinemia may affect the metabolic transformation of BCAAs in the FB.

In the AM group, the levels of His, Phe, Thr, Glu, Met, and Gly were all abnormal. Abnormalities of single amino acids in circulating blood are closely related to the prevalence of diseases such as histidinemia (Rojas et al., 2012), hyperphenylalaninemia and hyperprolinemia (Pontoizeau et al., 2016), as well as other amino-acid-related metabolic diseases. These results suggest that the amino acid composition in the hyperproteinemic silkworm FBs were altered, implying that they have adverse effects on tissues and their related functions.

Furthermore, the GPT and GOT activities in the FBs of the AM group changed significantly compared with those of the CK group (Figure 5C). At 24–192 h after the wandering stage, the activities of GPT and GOT both decreased in the CK group, and this was followed by an increase during the dissociation of the larval FB and the appearance of the adult FB. This can be interpreted as a physiologically adaptive change of silkworms during their metamorphosis. During this period, there was no significant change in the GPT activity in the FB tissues of the AM group, whereas the GOT activity increased and then decreased, which was opposite of that in the control group. These results suggest that hyperproteinemia interferes with amino acid transformations in the silkworm FB.

### Abnormal mechanisms of remodeling in the FBs of hyperproteinemic silkworms

**Figure 6:**
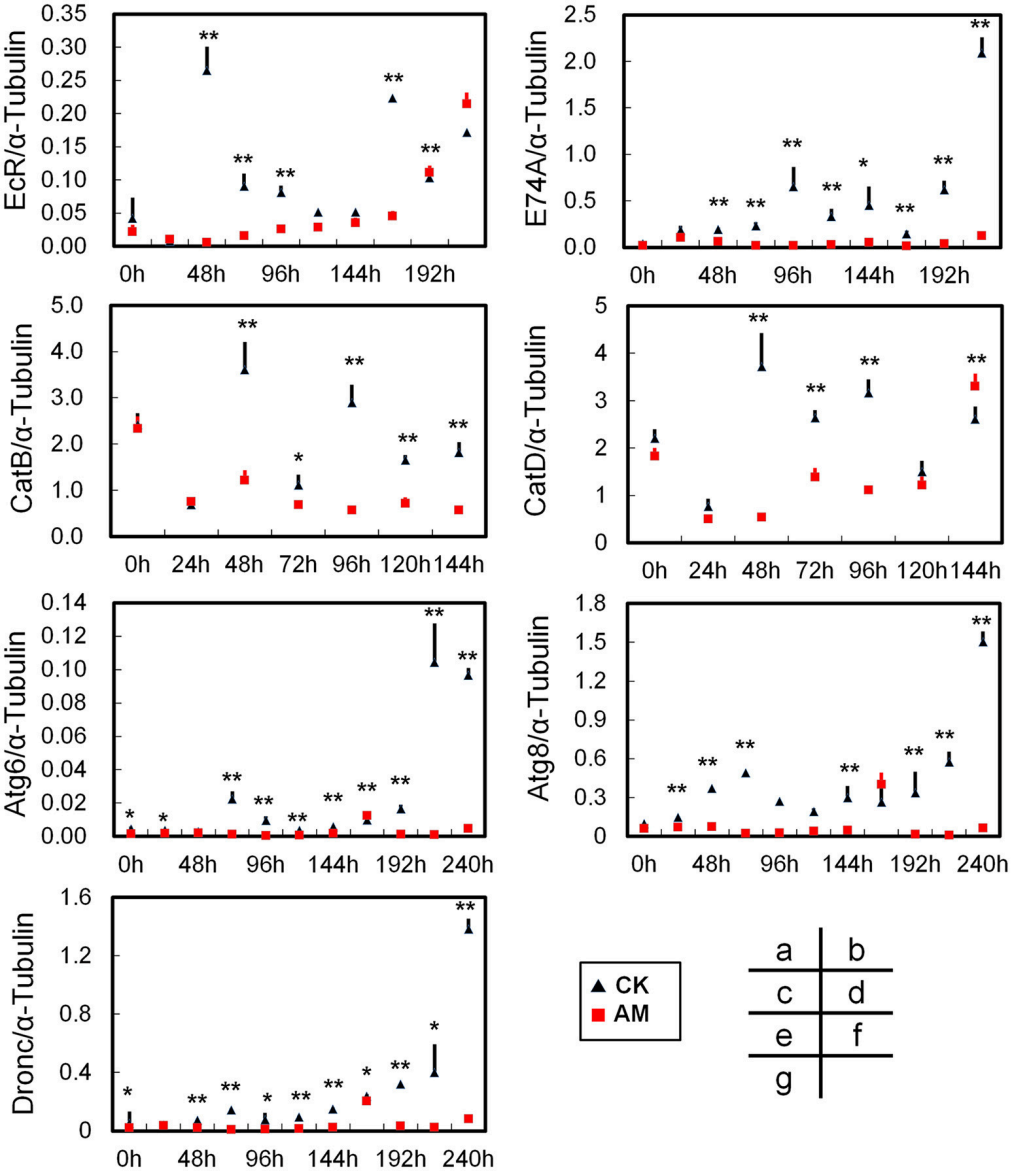
Quantitative real-time reverse transcription–PCR analysis of the relative transcript levels of the *BmEcR* **(A)**, *BmE74A* **(B)**, *BmCatB* **(C)**, *BmCatD* **(D)**, *BmAtg6* **(E)**, *BmAtg8* **(F)**, and *BmDronc* **(G)** genes in the FB during metamorphosis. Total RNA was isolated from each FB sample, which was a mixture from three individual silkworms at 24, 48, 72, and 96 h after hyperproteinemia was induced. Transcript levels of the *B. mori* α-tubulin gene were used as an internal reference. CK, the control group; AM, animal model of hyperproteinemia. The abscissa indicates the time points after the remodeling process, namely the time after the remodeling treatment. ^*^ and ^**^ indicate significant differences between the two groups at *P* < 0.05 and *P* < 0.01, respectively (3 samples were measured in each group, *n* = 3).

FB tissue remodeling undergo an ecdysone-mediated programmed cell death (PCD) pathway during metamorphosis (Tsuzuki et al., 2001; Gui et al., 2006; Sekimoto et al., 2006, 2007; Lee et al., 2009). To understand the molecular mechanism of hyperproteinemia that leads to abnormal FB remodeling in silkworms, we first investigated the transcriptional changes of key genes that are downstream of the ecdysone signaling pathway that affect FB dissociation. Although the *BmEcR* gene's transcript levels in the AM and CK groups were up-regulated from 72 to 216 h after hyperproteinemia was induced (Figure 6A), the *BmEcR* mRNA level in the AM group was significantly lower than in the CK group during the first 168 h of FB remodeling. At 216 h, the *BmEcR* mRNA level in the CK group was down-regulated, while in the AM group, the *BmEcR* transcriptional level was up-regulated. Correspondingly, the transcriptional level of the ecdysone-activated *BmE74A* gene was consistently and significantly lower in the AM group than in the CK group (Figure 6B). Thus, hyperproteinemia leads to a delay in the initiation of the ecdysone signaling pathway in FB tissues during silkworm metamorphosis.

**Figure 7:**
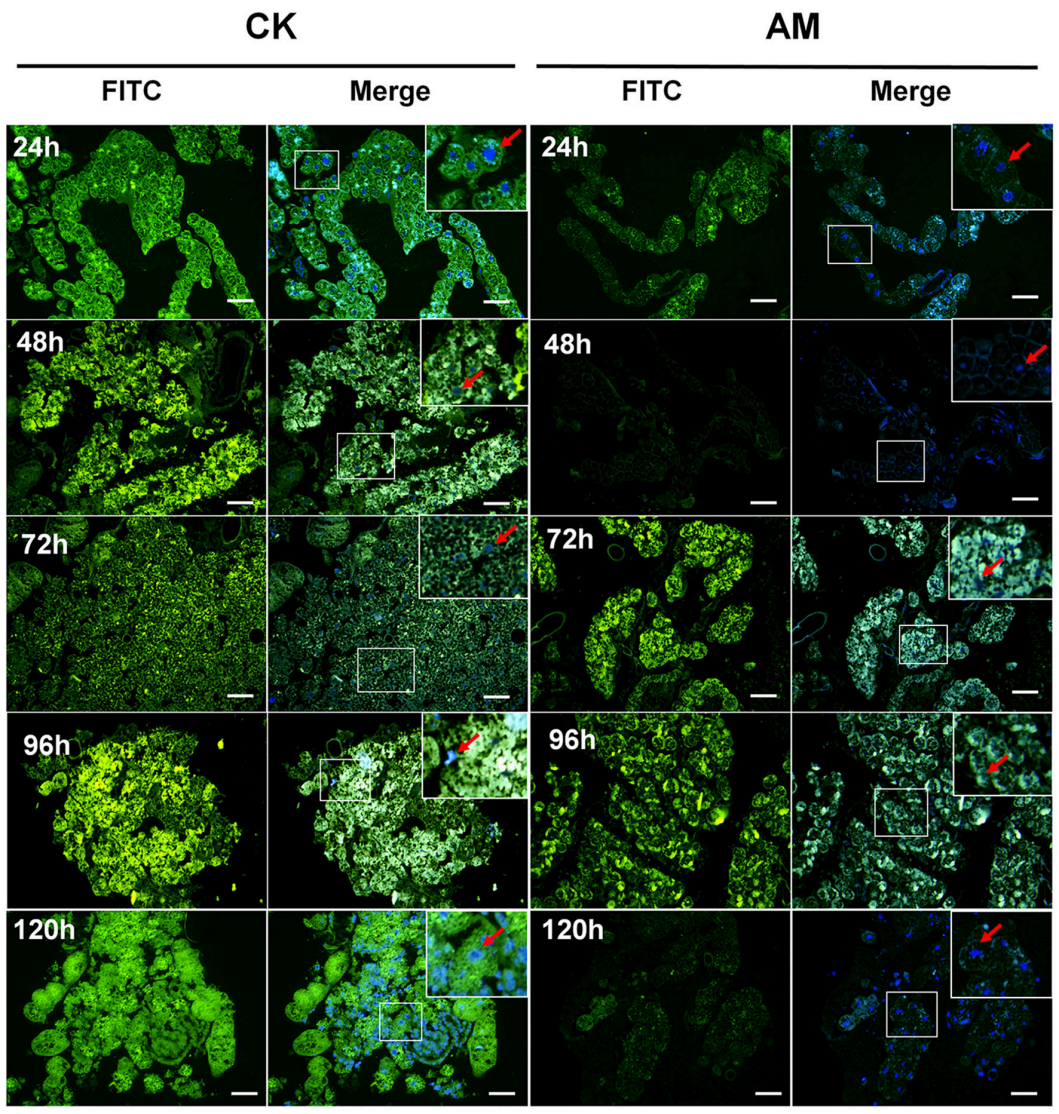
Localization of caspase-3 in fat body (FB) cross-sections at the pre-pupa stage. *Bombyx mori* larvae were treated and sampled as described in the Figure 1. CK and AM indicate the control and animal model groups, respectively. Cross-sections of the FB were dissected from silkworms at 24, 48, 72, 96, and 120 h after hyperproteinemia was induced. Caspase-3 (green fluorescence) was stained by an anti-caspase-3 antibody conjugated with fluorescein isothiocyanate (FITC). Merge, merged images for FITC and genomic DNA labeled with 4′,6-diamidino-2-phenylindole (DAPI). The red arrows represent the nucleus positions of fat body cells. The bar = 20 μm.

The transcriptional levels of the tissue dissociation-related *BmCatB* (Figure 6C) and *BmCatD* (Figure 6D) genes of FB cells were significantly lower in the AM group than in the CK group from 48 to 120 h after hyperproteinemia was induced. At 144 h after hyperproteinemia was induced, the transcriptional level of *BmCatD* in the AM group was higher than that in the CK group, but the transcriptional level of *BmCatB* was still low. Correspondingly, the transcriptional levels of the *BmAtg6* and *BmAtg8* genes (Figures 6E,F), as well as that of the *BmDronc* gene (Figure 6G), were consistently lower in the AM group compared with in the CK group during the first 240 h after hyperproteinemia was induced. The immunohistochemistry showed that at 24–96 h after hyperproteinemia was induced, the caspase-3 protein level in the AM group was lower than in the CK group, and the caspase-3 protein level in the AM group decreased significantly at 120 h, while it remained high in the CK group (Figure 7).

Furthermore, the staining of lysosomes that are involved in apoptosis induction showed that the intensity of red fluorescence was greater in the CK group than in the AM group (Figure S4). Almost all the FB cells in the CK group (48–72 h) showed strong fluorescence. At 96 h after hyperproteinemia was induced, the fluorescence in the AM group was still scattered in all the FB cells, while in the CK group, only a few FB cells showed strong, red fluorescence, and most cells showed no or weak red fluorescence. These results show that there were only a few cells still undergoing apoptosis and that new cells had emerged in the CK group, while in the AM group, the cells were still undergoing apoptosis.

The foregoing results consistently show that after hyperproteinemia was induced, the FB showed signs of apoptosis and autophagy disorders, resulting in the impairment of FB remodeling.

**Figure 8:**
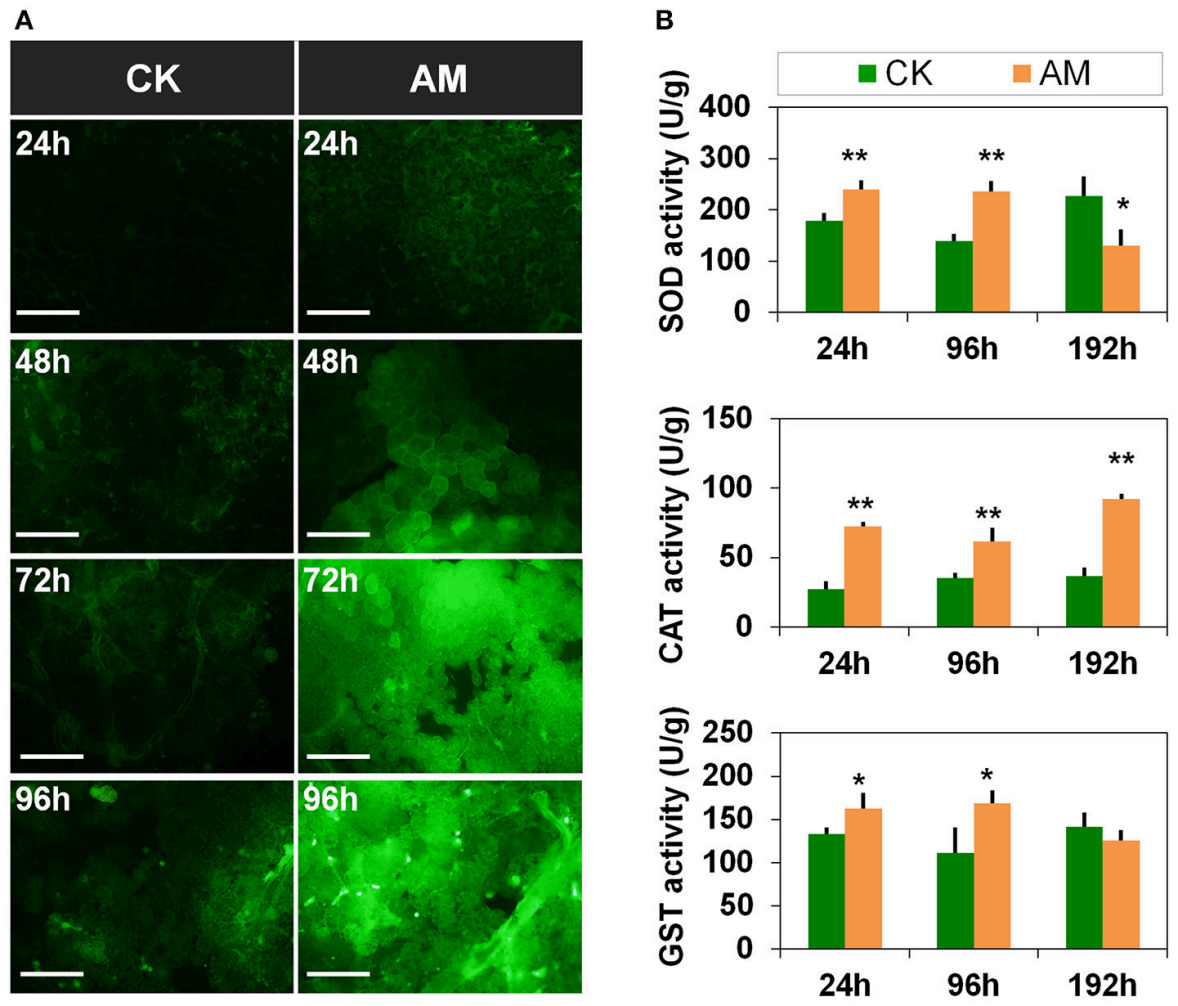
Changes in the oxidative stress level of the fat body (FB) during metamorphosis. *Bombyx mori* larvae were treated and sampled as described in the Figure 1. **(A)** Reactive oxygen species (ROS) staining of the FB. **(B)** Activities of super oxide dismutase (SOD), catalase (CAT), and glutathione S-transferase (GST) at 24, 48, 72, and 96 h after the induction of hyperproteinemia. Each FB sample, which consisted of a mixture from 10 silkworms, was obtained in triplicate and used for repeat measurement of SOD, CAT, and GST activity levels. ^*^ and ^**^ indicate significant differences between the two groups at *P* < 0.05 and *P* < 0.01, respectively (*n* = 3). The bar = 50 μm.

To investigate other possible causes of apoptosis and autophagy barriers in the FB, we investigated the effect of hyperproteinemia on FB oxidative stress. The ROS levels (Figure 8A) of the FBs in the AM group were significantly higher than in CK group from 24 to 96 h, especially at 72 h and 96 h, after hyperproteinemia was induced. The relative enzyme activities (Figure 8B) showed that the SOD activity in the AM group was significantly higher than in the CK group from 24 to 96 h, indicating that the FB had started to adapt to the oxidative stress caused by hyperproteinemia. However, at 192 h, the SOD activity level in the FBs of the AM group was significantly lower than that in the CK group, which may be related to FB damage. The CAT and GST activities also trended higher in the AM group, indicating that the FBs of silkworms in the AM group were decomposing the peroxides produced by SOD through the increased CAT activity. The increased GST activity level in FBs could help perform detoxification functions. However, at 192 h, the GST activity in the FBs decreased in the AM group, likely owing to FB damage, suggesting that the FB of silkworms during metamorphosis was under a high level of oxidative stress in response to the remodeling process, which resulted from cell autophagy and apoptosis, as well as other physiological changes. However, in the FB cells of the AM group, although the autophagy and apoptosis levels were lower than those of the CK group, hyperproteinemia caused a higher level of oxidative stress, which might be a further cause of the FB cell damage in the AM group.

### Endocrine hormone mitigates the hyperproteinemia-induced impairment of FB remodeling

**Figure 9:**
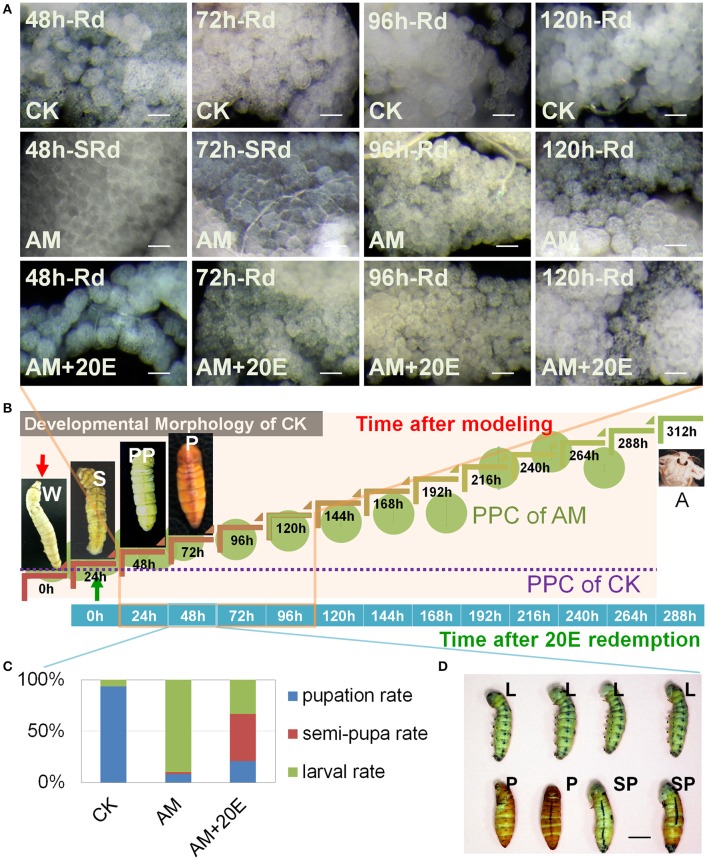
Influence of exogenous 20 (20E) on the hyperproteinemia-induced delay of fat body (FB) remodeling. **(A)** Typical histomorphology of the FB during larval–pupal metamorphosis. SRd, start of FB remodeling. Rd, FB remodeling. **(B)** Timeline of paurometabolic development from the wandering stage to the adult stage. The plasma protein concentration (PPC) throughout development, as shown in Figure 1B. Each hemolymph sample for surveying PPC consisted of an equal volume of hemolymph from three individual silkworms and was measured three times (*n* = 3). **(C,D)** Silkworm metamorphosis. Indices of development expressed in terms of the pupation, semi-pupa, and larval rates in the CK (*n* = 32 individuals), AM (*n* = 50 individuals) and AM+20E (*n* = 50 individuals) groups. CK, the control group; AM, animal model of hyperproteinemia in which hyperproteinemia was induced at the wandering stage; AM+20E, the larvae of the AM were saved by injecting individual larvae with 4 μg of 20E 24 h after the induction of hyperproteinemia; W, the wandering stage; S, the spinning stage; PP, the pre-pupal stage; P, the pupal stage; A, the adult stage; SP, the semi-pupa stage. L, the larval stage before the pre-pupal stage. Bars are 50 μm and 1 cm in **(A,D)**, respectively. To observe the morphology of the silkworm, the cocoon shells in the CK group were peeled off every 12 h until 48 h after spinning. This treatment also ensured the consistency of the micro-environment among the silkworms that spun their cocoons in the CK group and those that did not in the AM and AM+20E groups.

The foregoing results show that hyperproteinemia may result in weakened effects of ecdysone signaling during silkworm metamorphosis, leading to FB remodeling impairment due to cell autophagy and apoptosis, which may be associated with oxidative stress abnormalities. To further validate this hypothesis, we investigated the mitigating effect of 20E, an active component of ecdysone, on FB remodeling. The morphological observations of FBs showed that their morphology in 20E-treated group was almost synchronous with the normal FB at 48 h after the 20E injection (72 h after the induction of hyperproteinemia) (Figures 9A,B) and that the FB remodeling rate of the 20E-treated group was significantly faster than that of the AM group, indicating that the hyperproteinemia-induced impairment of FB remodeling and development were mitigated by 20E. However, 72 h after the 20E injection (96 h after the induction of hyperproteinemia), the ability of 20E to mitigate the hyperproteinemia-mediated impairment of FB development decreased remarkably, as indicated by the impairment level being the same as in the AM group (Figure 9A). The rate of metamorphosis at the individual level also showed that the hyperproteinemia-induced slowing of metamorphosis was effectively mitigated within 60 h after the injection of 20E (84 h after the induction of hyperproteinemia). The rate of semi-pupa and pupation in the 20E-treated group was 6.7-fold higher than that in the AM group (Figures 9C,D and Figure S5), and the mitigation effect was 71% compared with the CK group. It is worth noting that the rate of metamorphosis in the 20E-treated group decreased rapidly 72 h after the injection of 20E (96 h after the induction of hyperproteinemia), and that there was no significant difference compared with that in the AM group. Furthermore, in later generations, the mortality in the 20E-treated group was higher than that in the AM group (Figure S5).

The transcriptional levels of the *BmEcR* and *BmE74A* genes, which are representatives of the ecdysone signaling pathway, were significantly up-regulated at 24–96 h and 72 h, respectively, after the 20E injection (Figures S6a,b, respectively). The transcriptional levels of the tissue dissociation-related *BmCatB* and the autophagy signal pathway-related *BmAtg6* genes also significantly increased after 24 h, which was followed by a subsequent decrease and another increase at 96 h (Figures S6c,e, respectively). The expression levels of another tissue dissociation-related *BmCatD*, the autophagy signal pathway-related *BmAtg8*, and the apoptosis pathway-related *BmDronc* genes was also down-regulated at the early stage of development and significantly up-regulated at the middle and late stages after the injection of 20E (Figures S6d,f,g). The results show that 20E supplementation after the induction of hyperproteinemia rapidly up-regulated the transcriptional levels of *BmAtg6* and *BmCatB*, once the ecdysone signaling pathway in the FB cells was activated. This can further induce the expression of autophagy- and apoptosis-related genes during the middle and late developmental stages, as well as the expression of *BmCatD*.

## Discussion

Different from hyperlipidemia and hyperglycemia and other metabolic diseases, the clinical manifestations of hyperproteinemia are mainly severe liver and kidney diseases (Bergstedt and Lingen, 1957; Schneiderbaur, 1958; Salvesen, 2009), myeloma or angeioma (Foord, 1935; Gutman et al., 1941; Sonnet et al., 1961), severe infection (Adeyeba, 1985; Manning, 1998a; Magid, 2006; Riemer et al., 2016), or metabolic poisoning (Wang et al., 1986) caused by concurrent metabolic diseases. Therefore, it is difficult to distinguish the effects of primary diseases and hyperproteinemia on tissues and organisms. Animal disease models are great platforms for studying the pathogenesis of metabolic diseases, developing drugs for disease prevention, and clinical treatment (Berger, 2009). Unfortunately, there are no reports of hyperproteinemia disease models. It is envisioned that the establishment of a reliable hyperproteinemia model will have a positive impact on the evaluation of the effects of hyperproteinemia on tissues and organisms, as well as on the development of a corresponding treatment regimen for different primary diseases. In this study, we selected an invertebrate model animal, the silkworm, and constructed a hyperproteinemia disease model. This model has the following characteristics.

### No adverse effects of primary disease

The hyperproteinemia disease model constructed in this study makes use of the normal physiological process of silkworm metamorphosis, without the impacts of primary hyperproteinemia, and, therefore, it is closer to other metabolic disease models, such as those for hyperlipidemia and hyperglycemia. The degeneration and dissociation of the SG during metamorphosis can result in a transient physiological rise of the PPC, which, however, has been adapted during the long-term evolution of the silkworm. The PPC can return to a pre-elevated level 24 h after the transient physiological rise of the PPC. The silkworm hyperproteinemia model established in this study only increased the synthesis of non-harmful silk proteins, with a consistent structure and composition, in the silkworm, which induced a higher PPC during the remaining lifetime of the entire generation.

The degradation of the silkworm SG was almost synchronized with FB remodeling. The dissociated proteins released from the SG tissue by PCD are decomposed into amino acids in the hemolymph for further oxidative decomposition or transformation (Jia et al., 2007). The FB is a monolayer of cell tissue, and almost all FB cells directly contact the circulating hemolymph. Therefore, the FB sensitively reflects the hyperproteinemia-induced metabolic abnormalities in damaged visceral tissue. FB remodeling during metamorphosis occurs mainly through PCD, and mature FB cells differentiate from the primordial cells, which is highly similar to the regeneration and repair of mammalian tissue (Kaneko et al., 2011). In contrast, the FB has similar metabolic and detoxifying effects as the mammalian liver, which plays an important role in resisting oxidative stress (Dong et al., 2014; Fang et al., 2014; Yin et al., 2014). In the present study, our hyperproteinemia model based on SG degeneration was efficient and reasonable for studying FB remodeling.

In conclusion, the model of hyperproteinemia established in this study can be used to more reliably evaluate the damage of high PPC as well as the damage to the vitality of the organism.

### PPC can be controlled

The principle of this modeling approach is to control the PPC by regulating the levels of free silk proteins remaining in the SG of silkworm. Therefore, it is theoretically possible to establish a model in which the PPC is below the PPC of the model, but is higher than that of the control PPC, which is important for quantifying the effect of the PPC.

### Endocrine hormone mitigation is effective

The PCD of the silkworm, including metamorphic cells, is induced by the endocrine hormone ecdysone (Tsuzuki et al., 2001; Sekimoto et al., 2006). Ecdysone also induces the expression of key genes involved in hormone signaling, such as *BmEcR* and *BmE74A* genes (Sekimoto et al., 2006, 2007), as well as the synthesis of tissue-dissociating enzymes. Among them, cathepsin B and cathepsin D may be responsible for dissociating FB tissues in larvae, as well as during the metamorphosis of advanced lepidopteran insects (Xu and Kawasaki, 2001). The *BmCatB* expression during the sloughing of larvae, pupae, and adult silkworms can result in PCD (Lee et al., 2009), and *BmCatB* and *BmCatD* are highly expressed during insect larval metamorphosis. RNA interference of the two genes could block larval–pupal development (Gui et al., 2006; Lee et al., 2009).

The results of this study indicate that hyperproteinemia results in a decrease in the ecdysone signaling pathway and the level of lysosomes during the larval–pupal metamorphosis of silkworms, the down-regulation of *Atg6* and *Atg8*, which are key genes of the PCD signaling pathway, the down-regulation of the level of *BmDronc* (a key apoptosis pathway gene) transcription and the down-regulation of the level of the apoptotic capase-3 protein. The transcriptional levels of *BmCatB* and *BmCatD*, which play key roles during cell dissociation, were also lower than those of normal silkworm FB cells. It is noteworthy that the hyperproteinemia-induced blockage of metabolic FB remodeling and individual metamorphosis were mitigated by the exogenous active component of ecdysone, 20E. These results show that the hyperproteinemia AM constructed in this study is a metabolic disease model that is expected to be improved by endocrine hormones. This feature expands the scope for exploring the pathogenesis of hyperproteinemia, especially for developing hyperproteinemia-related drugs.

## Conclusions

In this study, an AM of hyperproteinemia was established in an invertebrate animal model, and it had a controllable PPC and no primary disease effects. This model demonstrates the pathological effects of hyperproteinemia on preventing the remodeling of FB by reducing endocrine hormones.

## Author contributions

S-QX and Y-HS conceived this project. X-DC, Y-LW, Y-FW, Q-YL, H-YM, and LW performed the research. X-DC, Y-FW, and S-QX analyzed the data and wrote the paper. All authors participated in the revision of this paper.

### Conflict of interest statement

The authors declare that the research was conducted in the absence of any commercial or financial relationships that could be construed as a potential conflict of interest.
