# Supplementary material for: Induced Hyperproteinemia and Its Effects on the Remodeling of Fat Bodies in Silkworm, Bombyx mori
Source: Front Physiol. 2018 Mar 29;9:302. doi: 10.3389/fphys.2018.00302 (PMC5884952; doi:10.3389/fphys.2018.00302)
Supplement: Supplementary file 1 [file DataSheet1.DOCX]

**An induced hyperproteinemia and its effect on the remodeling of fat body in silkworm, *Bombyx mori***

Xue-Dong Chen^1, 2†^ , Yong-Feng Wang^1,2†^ , Yu-Long Wang^1,2^, Qiu-Ying Li^1,2^, Huan-Yu Ma^1,2^, Lu Wang^1,2^, Yang-Hu Sima^1,2^, Shi-Qing Xu^1,2,^*

^1^ School of Biology and Basic Medical Sciences, Medical College, Soochow University, Suzhou 215123, China.

^2^ Institute of Agricultural Biotechnology & Ecology (IABE), Soochow University, Suzhou 215123, China.

^†^ These authors contributed equally to this work.

* Corresponding author. Email: szsqxu@suda.edu.cn.

**Supplementary Information**

**
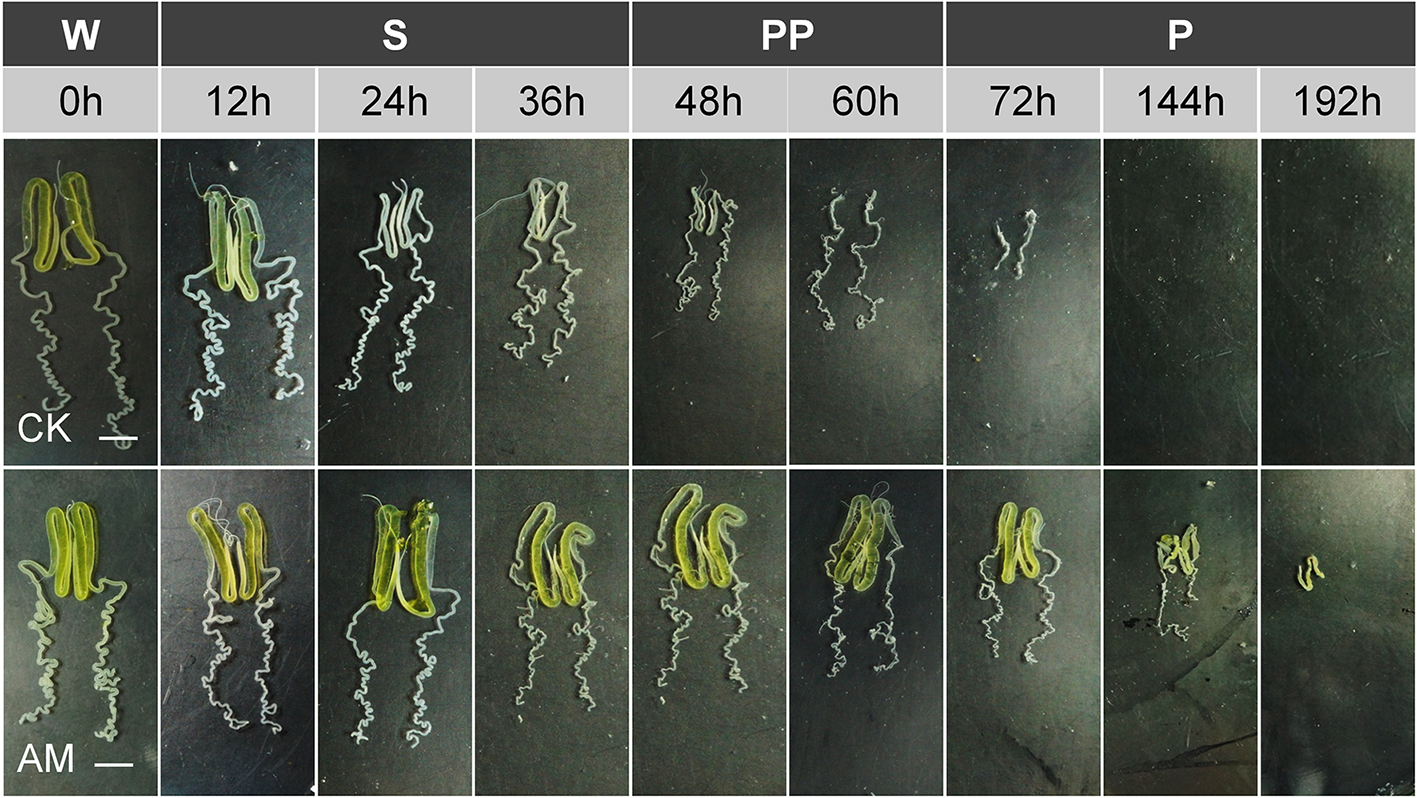
**

**FIGURE S1 Morphologic changes in *Bombyx* *mori*** **silk gland between the animal model of hyperproteinemia (AM) and its control group (CK).** Mature larvae of *B. mori* were treated and sampled as described in the Fig. 1 legend. W, the wandering stage. S, the spinning stage. PP, the pre-pupal stage. P, the pupal stage. The 0, 12, 24, 36, 48, 60, 72, 96, 144, and 196 h time points indicate the times after inducing hyperproteinemia. Bars are 0.5 cm.


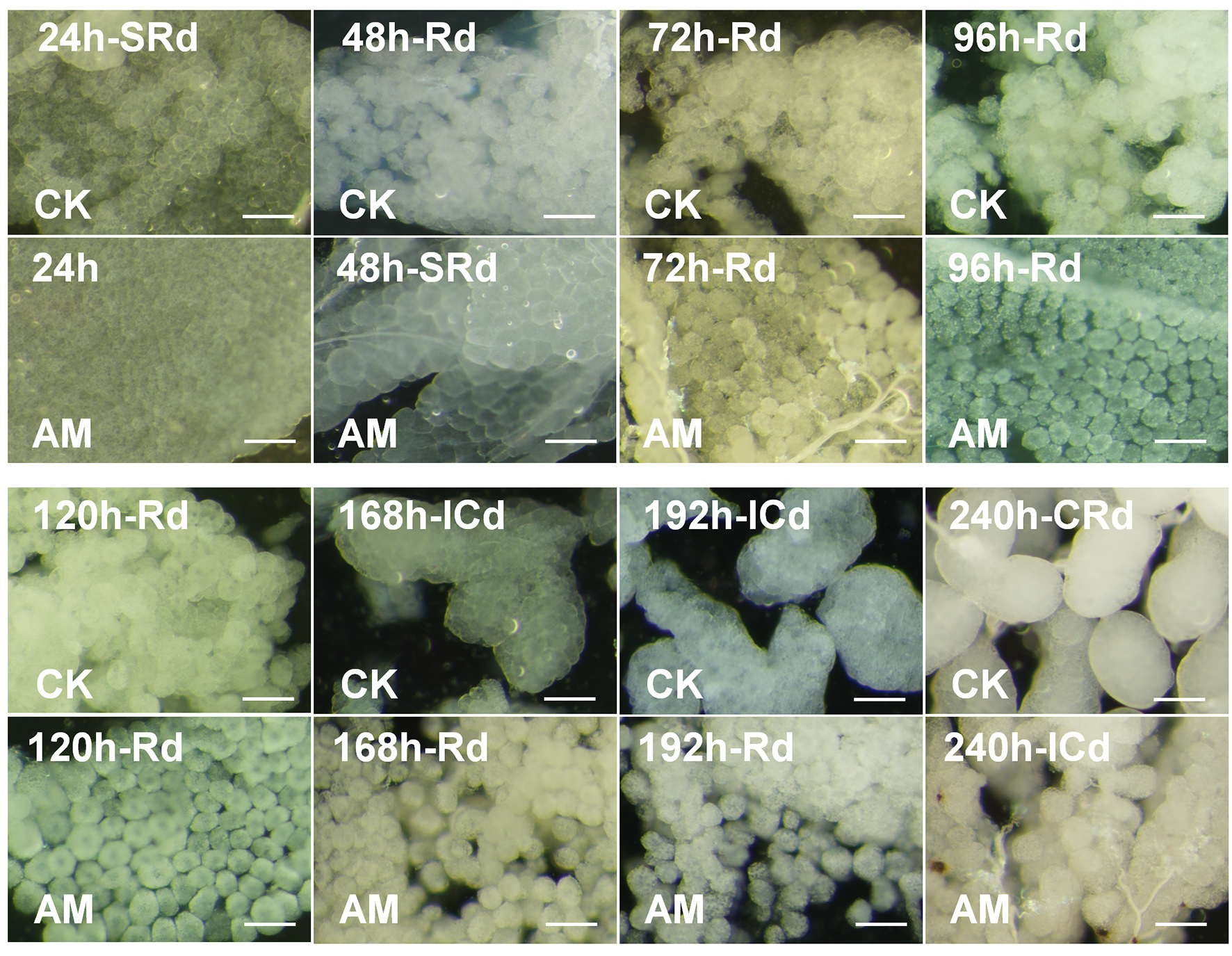


**FIGURE S2 Differences in morphology of the fat body between the animal model of hyperproteinemia (AM) and its control group (CK).** Larvae of *B. mori* were treated and sampled as described in the Fig. 1 legend. The spinneret was covered with paraffin wax for 24, 48, 72, 96, 120, 168, 192, and 240 h. SRd, start remodeling of the fat body. Rd, remodeling of the fat body. ICd, initially complete remodeling of the fat body. CRd, complete remodeling of the fat body. Bars are 20 μm.

**
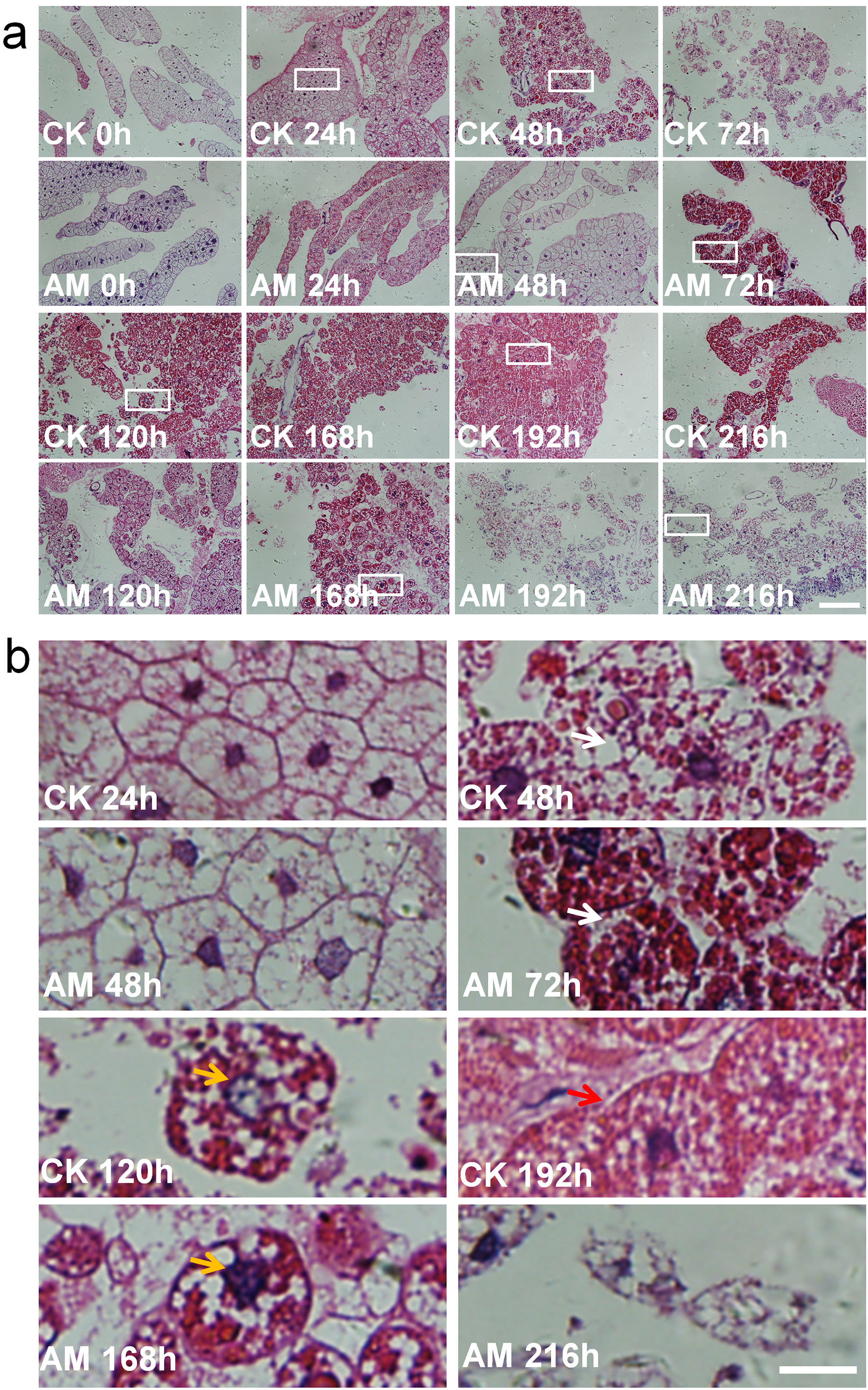
**

**FIGURE S3 Hematoxylin-eosin slice staining of the silkworm fat body.** *Bombyx mori* larvae were treated and sampled as described in the Figure 1 legend. CK indicates the control group, and AM indicates the animal model group. FB was analyzed at 0, 24, 48, 72, 120, 168, 192 and 216 h after inducing hyperproteinemia. Fig. S3b is a partial enlargement of the white boxes in Fig. S3a. White arrow shows vacuolation. The yellow arrow shows a complete nucleus. Red arrows show the tight junctions between fat body cells. The bar=50 μm in Fig. S3a; the bar=10 μm in Fig. S3b.


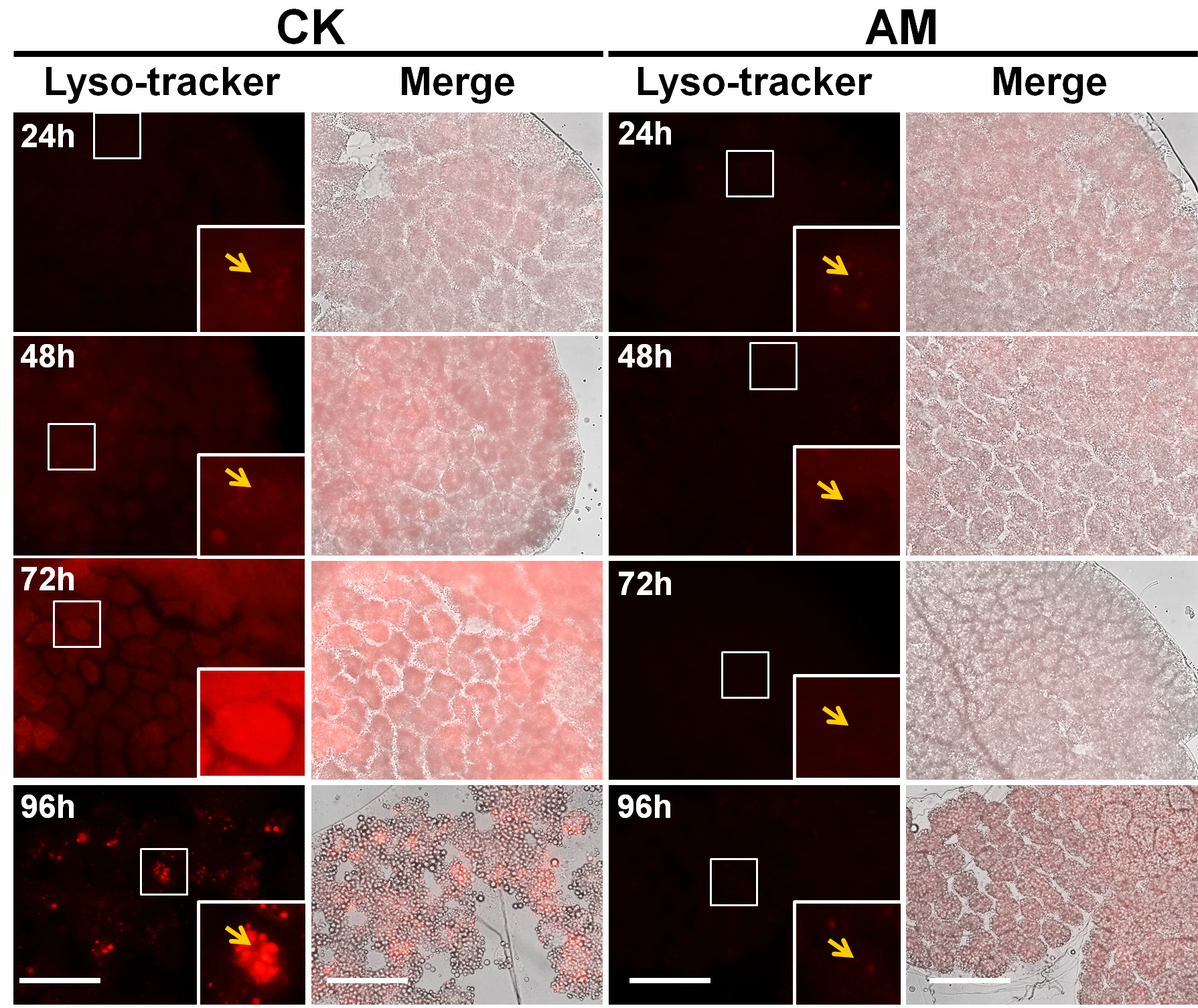


**FIGURE S4 Lysosome dyeing of the silkworm fat body.** Lyso-Tracker indicates lysosome dyeing; the merge is the superposition of lysosome dyeing and a bright field image. FB was analyzed at 24, 48, 72 and 96 h after inducing hyperproteinemia. The arrows point to the positions of nuclei. The bar=100 μm.


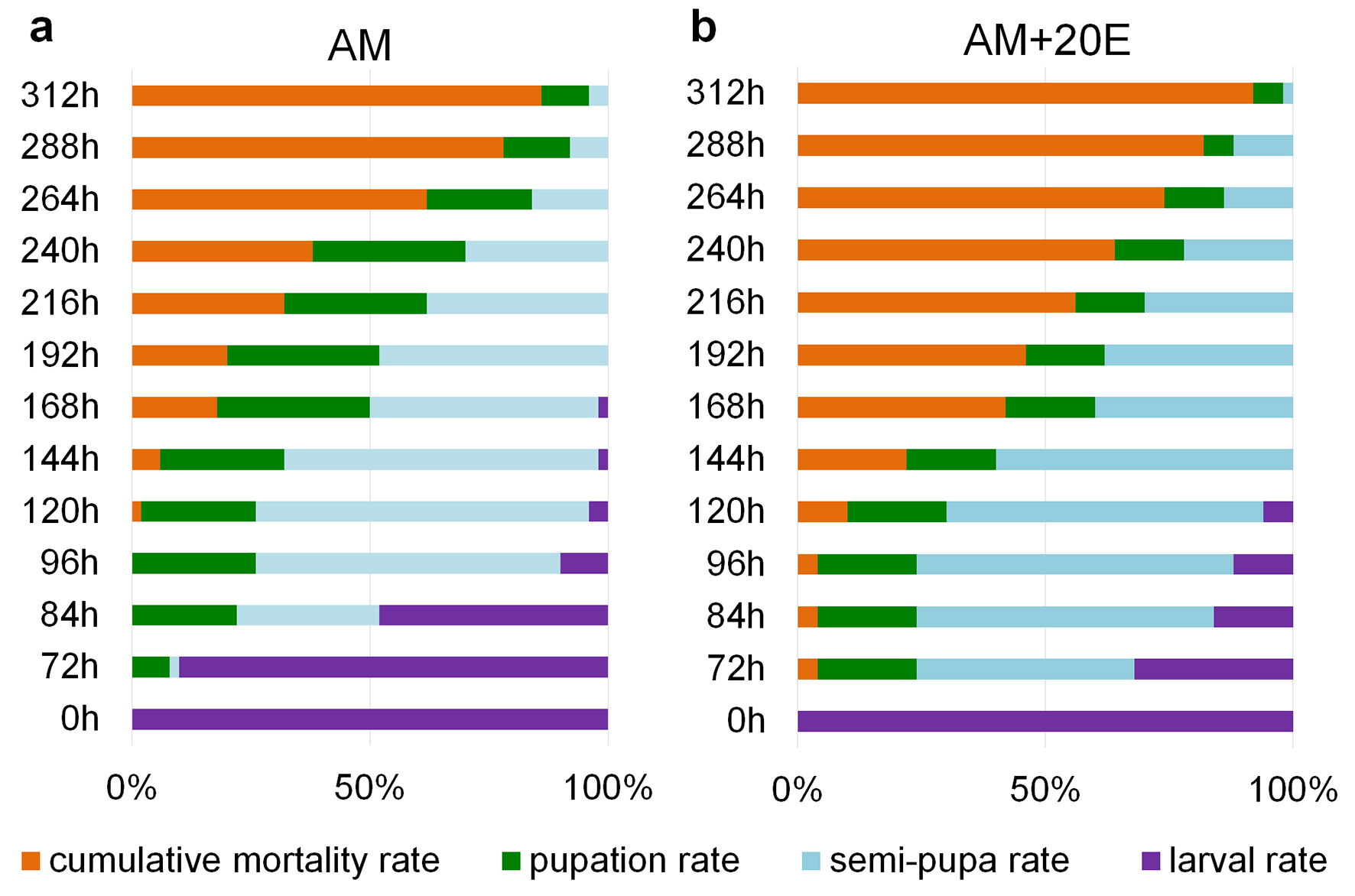


**FIGURE S5 Influence of exogenous hormone on silkworm development and survival.** Index of development and survival expressed in terms of the cumulative mortality, the pupation rate, the semi-pupa rate, and the larval rate in **(a)** the animal model of hyperproteinemia group (AM), in which modeling began at the wandering stage. (b) The larvae of AM (AM+20E) that were treated by injection with 4 μg of 20E 24 h after inducing hyperproteinemia. Index of development expressed in terms of the cumulative mortality, the pupation rate, semi-pupa rate, and larval rate in AM group (n=50 individuals) and AM+20E (n=50 individuals).

**
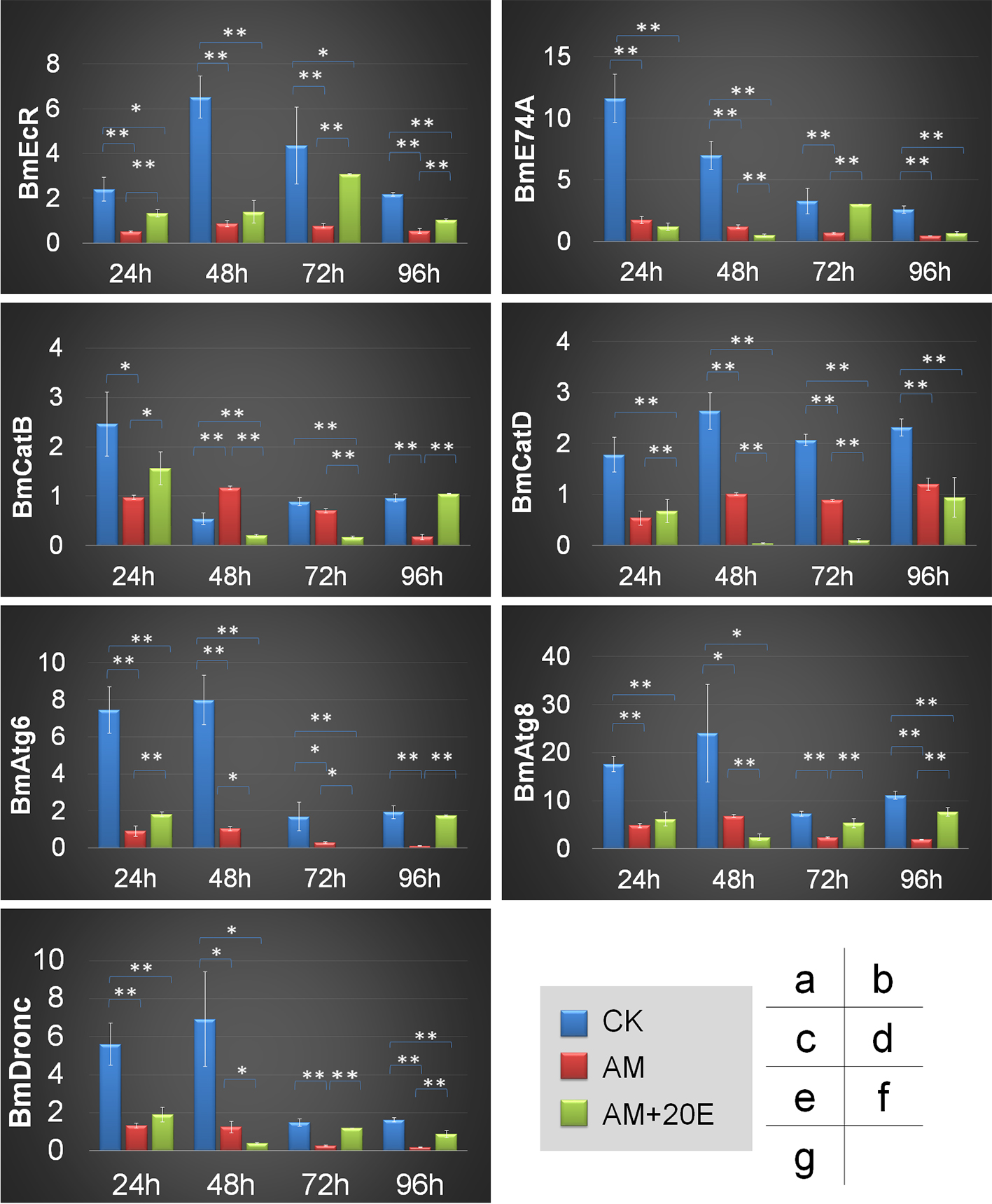
**

**FIGURE S6. qRT-PCR analysis of the relative transcript levels of the *BmEcR* (a), *BmE74A* (b), *BmCatB* (c), *BmCatD* (d), *BmAtg6* (e), *BmAtg8* (f), and *BmDronc* (g) genes in the fat body after the injection of exogenous 20-hydroxyecdysone (20E).** Total RNA was isolated from each FB sample collected from three individual silkworms at 24 h, 48 h, 72 h and 96 h after inducing hyperproteinemia. The transcript level of the *α-tubulin* gene was used as an internal reference. CK, the control group. AM, the animal model of hyperproteinemia. AM+20E, the larvae of the AM were treated by injection with 4 μg of 20E 24 h after inducing hyperproteinemia. * and ** indicate that the differences between the two groups reached the significant levels of *P*<0.05 and *P*<0.01, respectively (repeat measurement 3 times, *n*=3).

**TABLE S1 The Primers for qRT-PCR**

| Genes | Primers | Sequences |
| --- | --- | --- |
| Death regulator Nedd2-like caspase | *Dronc*-S | TGTGGCTGTCTTCCTTC |
|  | *Dronc*-A | ATCTAAGTCTGTGCCCTC |
| Autophagy 6 | *Atg6*-S | GTTATACGGTTCGGGTGG |
|  | *Atg6*-A | TGGAGTACGCATGTGGTG |
| Autophagy 8 | *Atg8*-S | AAGGCTAGGCTTGGAGAC |
|  | *Atg8*-A | CAGATGTGGGTGGAATGA |
| Cathepsin B | *CatB*-S | ACAAATGGCCTGACTGTCCA |
|  | *CatB*-A | AGGGATCTCGTAAGGTCTGC |
| Cathepsin D | *CatD*-S | GACACGTACTGGGAGTTCCA |
|  | *CatD*-A | CGTGTAGTACTTGCCGATGA |
| Ecdysone receptor (EcR) | *EcR*-S | CCACGATGCCTTTACCAATG |
|  | *EcR*-A | GTCGAGGTGCAGGACCTTTC |
| Ecdysone-induced protein 74a | *E74A*-S | ACCGCGTTCGACAACTTCGAT |
|  | *E74A*-A | CTGCAATCTTTGCTGCCCGTT |
| α-Tubulin | *Tub*-S | CTCCCTCCTCCATACCCT |
|  | *Tub*-A | ATCAACTACCAGCCACCC |

**TABLE S2** Amino acid content of fat body

| Time (h) | 24 | | 96 | | 192 | |
| --- | --- | --- | --- | --- | --- | --- |
|  | CK (μg/g) | AM (μg/g) | CK (μg/g) | AM (μg/g) | CK (μg/g) | AM (μg/g) |
| Asp | 76.47±0.79 | 76.32±0.49 | 76.42±0.43 | 75.90±0.63 | 81.06±8.45 | 78.53±1.20 |
| Glu | 56.48±17.11 | 60.93±14.50 | 84.18±10.36 | 69.23±3.19 | 121.83±35.27 | 49.21±8.83 |
| Ser | 21.73±5.66 | 19.59±0.54 | 26.59±7.97 | 32.28±5.78 | 71.47±22.56 | 46.80±9.79 |
| Cys-s | 0.26±0.22 | 0.30±0.07 | 0.17±0.13 | 0.11±0.19 | 0.32±0.48 | 0.26±0.26 |
| Met | 9.32±8.31 | 5.14±0.29 | 12.44±3.96 | 8.49±4.03 | 23.15±2.03 | 8.21±5.07 |
| Arg | 12.39±3.66 | 13.37±1.04 | 11.22±2.27 | 31.53±4.16 | 26.60±6.35 | 129.11±8.94 |
| Ile | 3.53±0.68 | 4.03±0.14 | 16.01±6.23 | 2.94±0.36 | 28.27±4.12 | 4.79±1.04 |
| Lys | 14.54±4.51 | 12.88±0.35 | 16.70±4.81 | 20.34±7.44 | 52.27±5.71 | 54.89±10.75 |
| Trp | 3.67±1.53 | 3.67±0.58 | 21.67±9.29 | 4.67±1.53 | 9.00±0.00 | 6.33±0.58 |
| His | 73.72±27.24 | 60.24±13.77 | 162.70±42.13 | 162.60±50.93 | 265.53±40.70 | 396.65±78.13 |
| Gly | 33.03±9.67 | 29.35±3.47 | 34.02±6.65 | 33.92±4.95 | 62.05±9.33 | 42.44±7.49 |
| Ala | 17.68±4.16 | 17.34±0.74 | 17.10±2.59 | 15.04±1.14 | 28.90±13.91 | 20.10±3.28 |
| Thr | 8.49±3.16 | 11.99±1.65 | 3.42±0.68 | 12.48±2.55 | 6.54±4.05 | 14.29±4.13 |
| Val | 11.92±7.55 | 8.08±3.91 | 13.68±4.99 | 8.54±1.66 | 31.76±5.77 | 12.42±3.23 |
| Phe | 29.51±0.62 | 29.13±1.54 | 40.41±28.36 | 28.36±2.26 | 47.19±2.98 | 31.97±1.63 |
| Leu | 8.40±0.88 | 8.28±0.16 | 23.54±9.30 | 6.60±1.88 | 32.06±4.29 | 8.45±2.66 |
| Pro | 12.34±4.75 | 9.01±0.78 | 12.01±4.30 | 13.62±5.81 | 40.25±6.87 | 38.73±8.20 |
| Tyr | 0.69±0.68 | 1.76±0.33 | 31.08±19.35 | 3.72±1.50 | 66.66±22.39 | 28.85±1.19 |

Note: Amino acid content of fat body (FB) were determined at 24 h, 96 h and 192 h after inducing hyperproteinemia. CK, the control group; AM, animal model of hyperproteinemia in which hyperproteinemia was induced at the wandering stage.
